# Evaluation of efficiency and safety of muscular region's acupuncture treatments for knee osteoarthritis: A protocol for systematic review and network meta-analysis

**DOI:** 10.1097/MD.0000000000026810

**Published:** 2021-08-06

**Authors:** Xiaoling Deng, Bingru Li, Xinju Hou, Xing Xu, Wei Xiong

**Affiliations:** Nanchang Hongdu Hospital of Traditional Chinese Medicine, Nanchang, Jiangxi Province, China.

**Keywords:** acupuncture, knee osteoarthritis, network meta-analysis, protocol

## Abstract

**Background:**

As a chronic degenerative disease, knee osteoarthritis (KOA) is mainly characterized by loose ligaments around the knee joint, degeneration of cartilage in the knee joint, and atrophy of surrounding muscles. According to related investigations, the incidence of knee osteoarthritis in China is 8.1%, of which 10.3% are women and 5.7% are men. Therefore, in order to improve the therapeutic effect of KOA, we must constantly explore new ways to treat the disease. The purpose of this study is to evaluate the effectiveness and safety of acupuncture with needle knife, blade needle, long-round needle, fire needle, micro-needle knife with conventional acupuncture intervention in KOA.

**Methods:**

Computer search of PubMed, Cochrane Library, Web of Science, CNKI, Wangfang, and VIP database, search for randomized controlled trials of muscular region's acupuncture therapy on KOA, the search time limit is to build the database until July 9, 2021. To ensure the comprehensiveness of the search, relevant references and conference literature are also included. After 2 researchers independently screened the literature, extracted data, and evaluated the risk of bias in the included studies, the Stata 14.0 software was used for data analysis.

**Results:**

The effectiveness and safety of muscular region's acupuncture in the treatment of patients with KOA will be systematically evaluated.

**Conclusion:**

The results of this study will provide strong evidence to determine whether muscular region's acupuncture is effective in the treatment of KOA.

**Registration number:**

INPLASY202170031 (https://inplasy.com/inplasy-2021-7-0031/)

## 1 Introduction

Knee osteoarthritis (KOA) is the most common bone and joint degenerative disease. Its main characteristics are laxity of ligaments around the knee joint, degeneration of cartilage in the knee joint, and peripheral muscle atrophy.^[1,2]^ It is also the main cause of lower limb pain and movement disorders in the elderly the reason. It is reported that the prevalence of symptomatic KOA in my country is 8.1%, and more than 65% of patients have intractable pain, which not only reduces the quality of life of patients, but also increases the burden of public health undertakings.^[3]^ The current treatment methods for KOA mainly include traditional Chinese medicine, acupuncture and massage, acupotomy, and modern medicine. The treatment of non-steroidal anti-inflammatory drugs, intra-articular injection and surgical treatment. Each treatment method has certain characteristics and curative effects, but which treatment method has the best curative effect has not yet been determined. The current treatment plan for knee osteoarthritis is drug intervention, such as analgesics and non-steroidal anti-inflammatory drugs, mainly to relieve symptoms and restore knee joint function.^[4,5]^ However, many adverse drug-related adverse events (AEs) including bleeding, gastric perforated ulcer ^[6]^ and increased risk of cardiovascular disease^[6]^ limit the use of these drugs. Therefore, non-drug treatments are becoming more and more common for doctors and patients of KOA.

Muscular region's acupuncture therapy was first recorded in “Lingshu·Jingjin”. Later physicians continued to invent and improve needles and acupuncture methods, which greatly enriched the connotation of it, including needle-knife, blade needles, round sharp needles and puncture methods. Acupuncture, which has been practiced for over 2500 years in China, is widely used to manage chronic pain. Health is attained through the flow of vital energy, Qi, through specific body paths called meridians, disease is caused by obstructions to this flow. In traditional Chinese acupuncture, needles are inserted at points along meridians to unblock these obstructions. Osteoarthritis is known as Bi syndrome in traditional Chinese medicine and acupuncture has been a standard treatment as early as the Song Dynasty. Acupuncture is one of the measures commonly used in clinical treatment of knee osteoarthritis. In recent years, many randomized controlled trials have confirmed its effectiveness.^[7–9]^

Network meta-analysis can combine direct evidence and indirect evidence to compare the efficacy of 3 or more interventions at the same time, and can rank the efficacy. Compared with traditional Meta analysis, it can be more comprehensive and comprehensive for comparison. The pros and cons of multiple interventions for 1 disease can provide high-level evidence-based evidence. At present, there are many kinds of muscular region's acupuncture treatments, and their effects are different. There is still a lack of comparative studies between different acupuncture methods. Therefore, this study adopted a network meta-analysis method, with conventional acupuncture as a common control, to compare the efficacy of various muscular region's acupuncture therapies on KOA, in order to screen the best acupuncture treatment plan and provide evidence-based reference for clinical treatment.

## 2 Methods

The protocol will be reported strictly according to the Preferred Reporting Items for Systematic Reviews and Meta-Analyses Protocols (PRISMA-P) statement. This study has been registered in the INPLASY website (registration number is INPLASY202170031).

### 2.1 Eligibility criteria of inclusion of studies

#### 2.1.1 Types of studies

We will include all randomized controlled trials that study the treatment of KOA in the muscular region's acupuncture. If the following conditions are included, they will be excluded.

The treatment group was combined with other treatment methods except for the 5 kinds of meridian acupuncture (needle-knife, blade needles, long-round needles, fire needles, micro-needle knives).

The data was obviously abnormal and the data could not be extracted.

Research and report of repeated substantive content signed by the same unit or period and the same author, choose one of them as the target document.

Non-RCT research: such as case reports, cohort studies, and cases Controlled studies, Meta analysis, experience summaries, reviews, animal experiments, etc.

Interventions incompatible with the inclusion criteria experiments.

#### 2.1.2 Types of participants

A patient diagnosed with KOA, in line with the diagnostic criteria for knee osteoarthritis of the American Academy of Rheumatology (1986, 1995, 2001), the diagnostic criteria for knee palsy (KOA) guidelines issued by the Orthopedics Branch of the Chinese Academy of Chinese Medicine, etc. Accepted KOA diagnostic criteria. There will also be no restrictions based on gender, race, and the course of the disease.

#### 2.1.3 Types of interventions

The treatment group was acupuncture with needle knife, blade needle, long-round needle, fire needle, micro-needle knife. The control group is a conventional acupuncture method or a comparison between the above 5 different, muscular region's acupuncture therapies.

#### 2.1.4 Types of outcome measures

At least 1 or more of the following outcome indicators are included: effective rate (Effective rate = [(recovery + obvious effect + effective)/total number of cases] × 100%), knee joint index score (The Western Ontario and McMaster Universities, WOMAC), Visual Analog Score (VAS) and Lysholm knee function score, and the trial data of the outcome indicators are reliable.

### 2.2 Electronic searches

A computer search for clinical randomized controlled trials of muscular region's acupuncture therapy for KOA. The Chinese database is searched by CNKI, Wangfang, VIP, and SinoMed. The foreign language database is based on PubMed, Web of science and Cochrane library. The search method is expanded subject headings (MeSH), and the search time limit is from the establishment of the database to July 9, 2021. In addition, reference documents are traced to supplement relevant documents to minimize omissions. The search will identify all randomized controlled trials to evaluate the effectiveness and safety of muscular region's acupuncture in the treatment of KOA. The search terms: “KOA” OR “knee osteoarthritis” OR “osteoarthritis of knee” OR “knee joint proliferative arthritis” AND “acupuncture” OR “needle” OR “needle-knife” OR “blade needle” OR “long circular needle” OR “acupotomy” OR “fire needle” AND “randomized controlled trial” OR “RCT”.

### 2.3 Data collection and analysis

#### 2.3.1 Literature screening and data extraction

**Figure 1:**
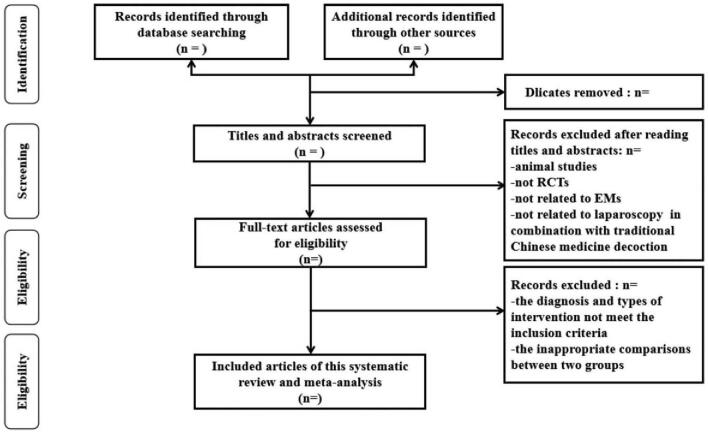
The research flowchart.

Import the documents extracted from the database into NoteExpress software for document management. First, eliminate duplicate documents, then quickly scan the titles and abstracts of the remaining documents to screen out those who do not meet the standards, and finally download the full text of the documents that may meet the requirements, read further, and filter out qualified literature. Two trained and qualified medical workers with clinical experience in orthopedics and acupuncture conduct literature retrieval, inclusion and exclusion, and the preliminary screening literature is obtained after verification, and then 2 researchers independently conduct data on the preliminary screening literature after extraction, it will be checked by the third researcher. If there is a disagreement, the third member and the author in charge will intervene and make a judgment. If there are missing data in the literature, email or phone the author of the article to obtain the data and supplement it. The flowchart will be demonstrated in Figure 1.

#### 2.3.2 Assessment of risk bias

The bias risk assessment tool will be evaluated based on the guidelines of the Cochrane Handbook for Systematic Reviews of Interventions, which is mainly carried out from 7 aspects: random sequence generation, allocation concealment, participants and personnel blinded, intentional analysis, completeness of the result data, selective reporting and other source bias; while the quality of the literature is evaluated using the quality evaluation tool Jadad scoring scale, independent of 2 researchers with GCP certificates. The evaluation will be conducted and checked by the third member. If there is a disagreement, the third member and the author in charge will intervene and make a judgment.

#### 2.3.3 Measures of treatment effect

Stata 15.0 software was used for statistical analysis of the data. The total effective rate is binary data, using odds ratio as the effect size; pain score (VAS) and functional score (WOMAC) as numerical variables, and the mean difference is used as the effect size. Each effect size is expressed in a 95% confidence interval, and the evidence network of each intervention is drawn. Predict the possible ranking probability of each treatment measure by drawing the surface under the cumulative ranking (SUCRA) graph. Finally use Revman5.3 software to draw a risk of bias chart to evaluate the risk bias of the included literature.

#### 2.3.4 Assessment of heterogeneity and sensitivity analysis

The *I*^2^ value is used to test the heterogeneity, and 50% (*I*^2^) and 0.05 (*P* value) are selected as the cut-off points. If the value shows small heterogeneity (*P* > .05, *I*^2^ ≤ 50%), the fixed-effects model is used for network meta-analysis. On the contrary, if there is heterogeneity (*P* < .05, *I*^2^ > 50%), use random effect model, and through subgroup analysis and sensitivity analysis to explore the source of heterogeneity, subgroup analysis based on the grouping includes treatment course, control group intervention measures, patient grouping plan, etc. If the source of heterogeneity or heterogeneity cannot be determined when the sex is too big, only do a descriptive analysis.

#### 2.3.5 Assessment of reporting biases

If the number of included studies for the outcome index is ≥10, Test the small sample effect and publication bias by drawing a “comparison-correction” funnel chart, take the effect size of each indicator as the abscissa and the standard error as the ordinate.^[10]^ If there is a difference in the symmetrical distribution, there will be publication bias or small sample effects.

### 2.4 Ethics and dissemination

Due to the agreement of the systematic review and meta-analysis in this article, all data in this study are from published studies and do not involve patient personal information, so ethics committee approval is not required. The results of this research will be distributed to peer-reviewed journals and published in relevant conferences.

## 3 Discussion

In fact, as meridian acupuncture is widely used in KOA, its effectiveness has been proven. There are now many directly compared meta-analysis literature studies that show the effectiveness of meridian acupuncture in the treatment of KOA.^[11–13]^ For example, Zhang^[12]^ et al conducted a meta-analysis study that showed that acupotomy can improve the effectiveness and reduce pain scores. Better than acupuncture. Wu^[13]^ et al conducted a meta-analysis that also showed that fire acupuncture can improve the symptoms of KOA better than conventional acupuncture and warm acupuncture. However, the above-mentioned meta-analysis of direct comparison does not have comparisons between meridian acupuncture therapies and cannot provide clear guidance for clinicians. Therefore, in this study, through the indirect comparison generated by the network meta-analysis, the curative effect comparison between the meridian acupuncture therapies was realized, and the advantages in the treatment of knee osteoarthritis were explored. In this study, a network meta-analysis was used to compare the curative effects of acupuncture with needle knife, blade needle, long-round needle, fire needle, micro-needle knife with conventional acupuncture intervention in KOA, so as to provide the effectiveness and Safety provides evidence-based medicine.

## Author contributions

**Conceptualization:** Xiaoling Deng, Xinju Hou, Xing Xu, Wei Xiong.

**Data curation:** Bingru Li, Xinju Hou, Xing Xu, Wei Xiong.

**Formal analysis**: Xiaoling Deng, Bingru Li, Xinju Hou.

**Funding acquisition:** Xiaoling Deng, Xing Xu, Wei Xiong.

**Investigation:** Bingru Li, Xinju Hou, Xing Xu.

**Methodology:** Xiaoling Deng, Bingru Li, Wei Xiong.

**Resources:** Bingru Li, Xinju Hou, Xing Xu, Wei Xiong.

**Software:** Xiaoling Deng, Bingru Li, Wei Xiong.

**Supervision:** Xinju Hou, Xing Xu, Wei Xiong.

**Validation:** Xiaoling Deng, Bingru Li, Xinju Hou.

**Visualization:** Xiaoling Deng, Xing Xu, Wei Xiong.

**Writing - original draft:** Bingru Li, Xinju Hou, Wei Xiong.

**Writing - review & editing:** Xiaoling Deng, Xinju Hou, Wei Xiong.

## References

[1] Yuan XL Meng HY Wang YC. Bone-cartilage interface crosstalk in osteoarthritis: potential pathways and future therapeutic strategies. Osteoarthritis Cartilage 2014;22:1077–89. 24928319 10.1016/j.joca.2014.05.023

[2] Tang X Wang S Zhan S. The prevalence of symptomatic knee osteoarthritis in China: results from the China health and retirement Longitudinal Study. Arthritis Rheumatol 2016;68:648–53. 26474054 10.1002/art.39465

[3] Vina ER Kwoh CK. Epidemiology of osteoarthritis: literature update. Curr Opin Rheumatol 2018;30:160–7. 29227353 10.1097/BOR.0000000000000479 PMC5832048

[4] Zhang W Moskowitz RW Nuki G. OARSI recommendations for the management of hip and knee osteoarthritis, Part II: OARSI evidence-based, expert consensus guidelines. Osteoarthritis Cartilage 2008;16:137–62. 18279766 10.1016/j.joca.2007.12.013

[5] Nelson AE Allen KD Golightly YM Goode AP Jordan JM. A systematic review of recommendations and guidelines for the management of osteoarthritis: the chronic osteoarthritis management initiative of the U.S. bone and joint initiative. Semin Arthritis Rheum 2014;43:701–12. 24387819 10.1016/j.semarthrit.2013.11.012

[6] Silverstein FE Faich G Goldstein JL. Gastrointestinal toxicity with celecoxib vs nonsteroidal anti-inflammatory drugs for osteoarthritis and rheumatoid arthritis: the CLASS study: a randomized controlled trial. Celecoxib Long-term Arthritis Safety Study. JAMA 2000;284:1247–55. 10979111 10.1001/jama.284.10.1247

[7] Lin LL Li YT Tu JF. Effectiveness and feasibility of acupuncture for knee osteoarthritis: a pilot randomized controlled trial. Clin Rehabil 2018;32:1666–75. 30037276 10.1177/0269215518790632

[8] Li J Li YX Luo LJ. The effectiveness and safety of acupuncture for knee osteoarthritis: an overview of systematic reviews. Medicine (Baltimore) 2019;98:e16301. 31305415 10.1097/MD.0000000000016301 PMC6641846

[9] Tu JF Yang JW Shi GX. Efficacy of intensive acupuncture versus sham acupuncture in knee osteoarthritis: a randomized controlled trial. Arthritis Rheumatol 2021;73:448–58. 33174383 10.1002/art.41584

[10] Sutton AJ Duval SJ Tweedie RL Abrams KR Jones DR. Empirical assessment of effect of publication bias on meta-analyses. BMJ 2000;320:1574–7. 10845965 10.1136/bmj.320.7249.1574 PMC27401

[11] Shu Q Ji LH Wang T Wang CJ Yang X Guo CQ. Meta-analysis of comparison of curative effect of meridian acupuncture and conventional acupuncture on knee osteoarthritis. Modernization of Traditional Chinese Medicine and Materia Medica-World Science and Technology 2020;22:876–84.

[12] Zhang L Wei MB Liu AF. Meta-analysis of the clinical efficacy of small needle knife and acupuncture in the treatment of knee osteoarthritis. Tianjin Traditi Chin Med 2019;36:253–7.

[13] Wu HZ Shi PP Wang JM. Meta-analysis of the therapeutic effect of fire needling on knee osteoarthritis. Chin J Information Traditi Chin Med 2014;21:10–4.

